# Gluten-Free Products: Do We Need to Update Our Knowledge?

**DOI:** 10.3390/foods11233839

**Published:** 2022-11-28

**Authors:** Claudia Mármol-Soler, Silvia Matias, Jonatan Miranda, Idoia Larretxi, María del Pilar Fernández-Gil, María Ángeles Bustamante, Itziar Churruca, Olaia Martínez, Edurne Simón

**Affiliations:** 1 Gluten Analysis Laboratory, Department of Pharmacy and Food Science, University of the Basque Country (UPV/EHU), 01006 Vitoria-Gasteiz, Spain; 2 GLUTEN3S Research Group, Department of Nutrition and Food Science, University of the Basque Country, 01006 Vitoria-Gasteiz, Spain; 3 Bioaraba, Nutrición y Seguridad Alimentaria, 01006 Vitoria-Gasteiz, Spain; 4 Centro Integral de Atención a Mayores San Prudencio, 01006 Vitoria-Gasteiz, Spain

**Keywords:** celiac disease, gluten-free, diet, food, nutritional composition

## Abstract

The gluten-free (GF) food market has been growing exponentially in recent years. However, GF products could contribute to imbalanced diets. The main objective of the present work was to perform a temporal nutritional comparison of GF foods over the last nine years. At the same time, the nutritional compositions of 104 GF products currently marketed in Spain were assessed and compared with their gluten-containing counterparts. Diets including GF products and the equivalent diets with homologous products with gluten were compared as well, the subjects being 25 adult celiac patients. A comparison of GF products (GFPs) in 2013 and 2022 showed nutritional differences in the groups of cookies, breakfast cereals, doughs/puff pastries/pizzas, and breads. The pasta group stands out from the rest due to an important decrease in energy, protein, simple carbohydrates, saturated lipids, dietary fiber, and salt. Comparing GF and gluten-containing homologous products in 2022, the major differences were found in protein and salt. Accordingly, GFPs lead to a diet lower in protein and higher in carbohydrates. Despite improvements in the formulation of GF products in recent years, their macronutrient profile maintains marked variation, and they cannot be considered nutritionally equivalent to their gluten-containing counterparts.

## 1. Introduction

The incidence of celiac disease (CD) is currently increasing exponentially in every country. This fact is not only due to environmental factors that can decrease tolerance to gluten in the diet but also to improved diagnosis [1].

CD is a systemic immune-mediated disease caused by gluten and related prolamins in genetically susceptible individuals and is characterized by the presence of a variable combination of gluten-dependent clinical manifestations, enteropathy, specific antibodies, and HLA–DQ2 or HLA–DQ8 haplotypes [2]. Apart from CD, other gluten-related disorders (GRDs) include three major forms of gluten reactions, such as allergic, autoimmune, and possibly immune-mediated. Despite differences in pathological mechanisms, clinical manifestations, and epidemiology, the treatment of all GRDs consists in excluding gluten-containing cereals and byproducts from the diet, which affects almost 10% of the population [3].

During the last few decades, the uptake of gluten-free products (GFPs) has increased in the general population. This is probably due to a public perception that a gluten-free (GF) diet provides health benefits, and at present, GFPs are also widely used by people without any GRDs [4]. In this respect, the food industry needs to expand and diversify both in terms of progress in ingredients and formulations as well as in the production of functional foods [5]. A 2017 survey of American consumers of GF bread found that 31% do so as a lifestyle choice [6]. Likewise, a questionnaire in the United Kingdom showed that 3.7% claimed to follow a GF diet. A survey reported on National Public Radio found that almost one-third of adult Americans would prefer to reduce or avoid gluten consumption altogether while in certain groups, such as athletes, up to 50% reported variable adherence to a GF diet [7].

However, concerns have been raised in the scientific community regarding the health aspects and nutritional quality of GFPs [8]. Numerous publications have shown that people suffering from GRDs have nutritionally imbalanced diets, such as vitamin and mineral deficiency and inappropriate energy, fiber, or macronutrient intake. This situation is justified, in part, by the fact that specific products for this purpose are rarely fortified, unlike products for general use. In addition, some studies highlighted the low protein content and high fat and salt content of GFPs [9]. The most recent assessments on the nutritional quality of GFPs currently available on the market show key inadequacies—low protein content and high fat and salt content—compared to their homologous gluten-containing products (GCPs) [10]. In addition to nutrient deficiencies, other issues make it difficult for gluten products to be replaced. For instance, GF dough is more difficult to handle due to a lack of cohesiveness, elasticity, and baking quality. GFPs are usually characterized by high starch content, low fiber content, short shelf life, or texture issues like increased breadcrumb hardness. Within this aim, researchers are committed to finding formulations involving mixtures of different GF flours and ingredients to obtain products that are similar to wheat-containing products [11].

After a decade, an update on the measures adopted in the food industry in the manufacturing of these products and their efficacy seems pertinent. The main objective of this study was to conduct a temporary comparison of GF foods against their counterparts with gluten using the previous research data from 2013. Furthermore, this research seeks to compare the nutritional composition of 104 GFPs currently available in Spain with their homologous GCPs to assess eventual deviations in the composition of the foods and to contrast the diet including GFPs and its equivalent diet with homologous GCPs in 25 celiac adults. This analysis was carried out as a continuation of the same project published in 2013, where marked nutritional differences between GFPs with respect to their counterparts were revealed.

## 2. Materials and Methods

### 2.1. Study Design and Data Collection

Three descriptive and comparative studies were conducted. The first study compares the nutritional information panel (NIP) of a GFP from 2013 against the NIP of the same GFP currently available in 2022. The 2013 data were taken from the database used in a previous study [9]. Only 104 GFPs from 11 specific brands were commercially available from those studied in 2013. The 2022 data were collected using the same methods as in 2013. Briefly, between October 2021 and April 2022, NIP data were systematically obtained from all packaged GFPs available at the three biggest supermarkets in Vitoria-Gasteiz (Spain). When the same product was presented in different pack sizes, only one entry was recorded.

In the second study, a nutritional comparison was made between 104 currently available GFPs and their analogs with gluten. The comparison was also based on NIP, and this information was recorded as previously stated for GFP. The 104 gluten-containing analogs sampled for this study in 2022 belonged to 49 different brands.

For both studies, NIP was registered into a spreadsheet, and nutrient composition was analyzed for all the samples collected: total carbohydrates, simple carbohydrates, protein, total lipids, saturated lipids, fiber, and salt. Differences in micronutrient content, such as vitamins and minerals, were not studied. Using the reference of Miranda et al. [9], products were classified into the following groups: cookies (*n* = 16), pastries (*n* = 17), pastas (*n* = 6), breakfast cereals (*n* = 8), cereal bars (*n* = 2), baby foods (*n* = 7), flours (*n* = 3), dough/puff pastry/pizzas (*n* = 6), cakes (*n* = 1), breads (*n* = 34), and others (*n* = 4).

The third study highlighted the impact on nutritional value of a possible GFP inclusion in a celiac diet. With this aim, a comparison between the diet that includes GFPs and the same diet with gluten foodstuffs (a simulated diet) was carried out as described in the following subsections.

### 2.2. Subjects

Twenty-five celiac adults from Spain took part in the study (20 females and 5 males, aged between 27 and 60 years). Exclusion criteria included history of cardiovascular disease or diabetes, pregnancy, thyroid disorders, total cholesterol levels > 300 mg/dL, levels of triglyceride > 300 mg/ dL, and blood pressure level > 140/90 mm Hg. All participants received oral and written information about the nature and purpose of the survey, and all of them gave written consent for involvement in the study. This study was approved by Ethical Committee of The Basque Country (Code PI 2016069). All procedures followed were in accordance with the ethical standards of the responsible committee on human experimentation (institutional and national) and with the Helsinki Declaration of 1975 as revised in 2008.

### 2.3. Dietary Assessment

Three days of food records (two weekdays and one weekend day) were selected for each participant. A 24 h food recall and a food frequency questionnaire were filled in by each person. Food portions and amounts were determined using photographs, and nutrient intake was calculated by a computerized nutrition program system: GlutenFreeDiet [12].

A simulation of a gluten-containing diet was performed by duplicating recorded GF diets and replacing GF foodstuffs with their gluten-containing counterparts. Then, a nutritional comparison between both diets (GF and gluten-containing) was undertaken.

### 2.4. Statistical Analysis

Statistical analyses of the three studies were performed by using the IBM SPSS statistics 28 software (IBM Inc., Armonk, NY, USA). Results for continuous variables are given as the arithmetic mean ± SD and the range. After confirming the skewed distribution of variables, energy, nutrients, and fiber by the Shapiro–Wilk test, collected data were analyzed by the Wilcoxon test for paired samples. *p* values < 0.05 were accepted as statistically significant. The effect size (r) was calculated using the following formula: (r = z/√n) [13]. The effect size was interpreted based on the commonly used Cohen’s criteria of 0.10–0.29 for small effect, 0.30–0.49 for medium effect, and 0.50–1.0 for large effect [14].

## 3. Results and Discussion

### 3.1. Nutritional Composition of GFPs in 2013 and 2022

When comparing the GFPs of the year 2022 with 2013, significant general changes were found in the following variables: a decrease in protein (from 4.73 ± 3.09 in 2013 to 4.55 ± 3.08 in 2022; *p* = 0.025) and an increase in dietary fiber (3.27 ± 2.66 in 2013 to 3.78 ± 2.72 in 2022; *p* = 0.031). In order to analyze these general changes in depth, the energy, nutrients, and fiber data are presented in Table 1, organized by food groups.

From the food groups studied, pasta, cookies, breakfast cereals, doughs/puff pastries/pizzas, and breads demonstrated the most relevant fluctuations. The cookies group presented a significant decrease in the amounts of protein (−16.4%; *p* = 0.02). The effect size of this variation was medium (r = −0.43) while the rest of the variables did not reach the 0.3 threshold, which made their interpretation difficult. Breakfast cereals and breads showed a significant reduction in saturated lipids over time (−14.4% and −30.2% respectively). However, in the case of breakfast cereals, this alteration was just a tendency (*p* = 0.08), and for breads, the effect size was not high enough (r ≥ 0.10), which greatly restricted its extrapolation. By contrast, the doughs/ puff pastries/ pizzas group displayed a significant rise and a large effect on the content of dietary fiber (+39.7%; *p* = 0.04; r = −0.59).

Compared to the 2013 data, the pasta group stands out from the rest. Energy (−1.1%), protein (−7.2%), simple carbohydrates (−7.5%), saturated lipids (−44.1%), dietary fiber (−23.5%), and salt (−81.6%) contents decreased significantly (Table 1). In order to explain the nutritional variations observed in the pasta group over time, ingredients used to make all the GFPs classified as pasta (*n* = 6) were analyzed in both years, 2013 and 2022. Concretely, the foods’ ingredient information and order on their labels were taken into account. It is important to highlight that ingredients are listed in order of weight, beginning with the ingredient that weighs the most and ending with the ingredient that weighs the least.

**Figure 1:**
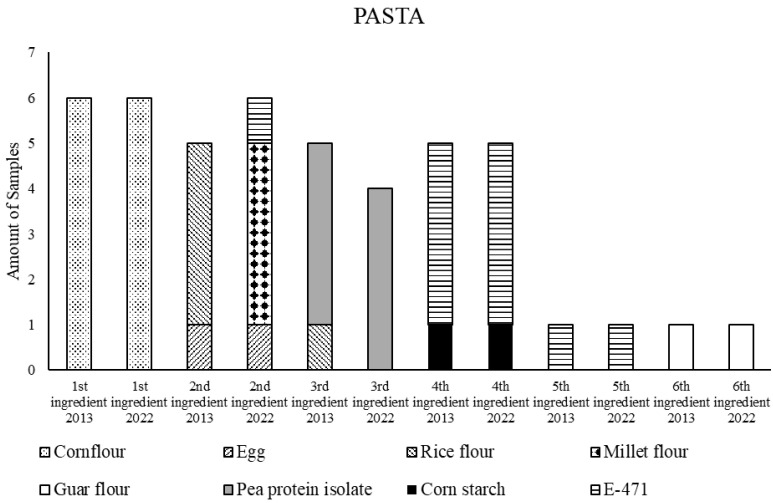
Comparison of the presence and weight of the ingredients used for GF pasta formulation from the most-used ingredient to the least in both 2013 and 2022. Amount of samples in which each ingredient is listed in this position is expressed according to the food label.

Figure 1 describes the amount of GFP in which each ingredient is included and its position, from the first to the sixth, in 2013 and 2022. The relevance of some ingredients for pasta foodstuff composition has not changed in almost a decade. These were the cases of corn flour (first position ingredient in the list), E-471 (fourth position and fifth position ingredients), cornstarch (fourth position ingredient), and guar flour (sixth position ingredient). Nevertheless, other ingredients have changed. For instance, in 2013 the second main ingredient in pasta was egg and/or rice flour. Conversely, nowadays, rice flour has been replaced by millet flour while the use of egg remains unchanged. In addition, E-471 has risen to the second ingredient in 2022. Likewise, rice flour appeared as the third ingredient in 2013 but not in 2022 anymore.

Checking the nutritional composition of millet and rice flours (USDA database), it was worth noting that millet flour had a higher fiber content compared to rice flour (3.5 g/100 g against 2.4 g/100 g). Consequently, our data displayed that the replacement of rice flour with millet flour in a pasta formula could have a direct contribution to its fiber content. This statement, achieved for the rest of the GF groups analyzed, was not reached in the pasta group, probably due to the limited number of samples available.

From a nutritional point of view, Culetu et al. [11] reported in their comparative study that GF millet flour presented a higher percentage of fiber content (7.79%) compared to GF rice flour (0.88%). These changes were similar to those observed in the current study, where fiber content improved after replacing rice flour with millet flour in the pasta composition, which could justify the improvement described from 2013 to 2022 in GF pasta. Moreover, in their analysis, millet recorded different protein values depending on the type used [11]: 11.9% for common millet flour, 8% for pearl millet flour, and 7.3% for finger millet flour. These variations could explain possible disparities in the protein content of pastas. Similarly, a recent study investigating the rheological and tribological properties of pastas obtained from various types of cereal grains showed that millet flours had higher fiber content compared to rice flours [15]. Additionally, in line with our results, they described that the percentage of protein content was higher in rice flour compared with millet flour.

The nutritional composition of flours did not justify the alteration that occurred in the rest of the parameters studied, such as energy, simple carbohydrates, saturated lipids, and sodium contents. Not only did the presence or the general relevance (position in the list) of concrete ingredients have an impact on nutritional variables, but it also affected the relative amount of each ingredient in the product recipe. However, as previously stated, ingredient lists of food labels just provided restricted information, so it is not possible to know the specific quantity of each ingredient included in the formulations. This implies an inability to measure the actual percentage in which each flour is incorporated. Given this circumstance, it was presumed that the substitution of rice flour for millet flour in the new pasta recipe was not complete. Pang et al. [15] noted that GF corn flour had a lower protein content (7.25%) compared to rice flour (8.15%). In the present study, corn flour remains the main ingredient of the pasta formulations followed by millet flour. Despite possible changes in ingredients and their percentage contribution to the final product, it could be considered that the relative amount of corn is currently higher compared to 2013, which justifies the diminution of protein.

Another relevant aspect to note is that the simplest way to improve the structure of GFPs is by adding other functional ingredients and additives (e.g., starches, protein, gum, hydrocolloids, emulsifiers, dietary fiber, etc.) to wheat flour substitutes [16]. The use of emulsifiers has increased in recent years due to their great capacity to improve the quality of pasta. Therefore, several ingredients are displayed as alternatives to gluten in order to create a network that can withstand the physical stresses of cooking and impart firmness to the product. Emulsifiers act as lubricants in the extrusion process and provide firmer consistency and a less sticky surface by controlling starch swelling and leaching processes during cooking, thereby improving the texture of the final product [17]. When comparing rice pasta (common GF pasta) with millet pasta in terms of texture parameters (starchiness, stickiness, and graininess), millet pasta has higher values according to the texture scoring system reported by others [18]. Subsequently, the substitution of rice flour with millet flour allows fewer emulsifiers to be added to the preparation. Regarding additive E-471 (mono- and diacylglycerols), its quantitative presence is not known in either of the years studied (2013 and 2022). Nevertheless, it can be assumed that this emulsifier is currently used in smaller quantities, which would explain the observed decrease in the amounts of lipids and energy in the pasta.

Nutrition labeling aims to help customers to improve their nutritional behavior. According to Perez-Armijo et al. [19], Nutri-Score is the most applied method in the European Union (EU), including Spain from 2021 on. According to a network meta-analysis by Song et al., (2021) [20], all color-coded and warning labels were significantly associated with changes in purchasing behavior and intention. These methods propose beneficial effects by encouraging the purchase of healthier products and reducing the purchase of less beneficial options. The overall nutritional quality of processed foods and drinks chosen tends to be higher when they have lower energy, sodium/salt, total fat, and saturated fat content. As a result, there is possible social pressure on the industrial stakeholders to improve their products to meet the expected standards, especially in the field of GF foods. Accordingly, the addition of Nutri-Scores would ease the identification of the nutritional quality, empowering consumers and possibly manufacturers as well to improve the nutritional quality of GFPs [21]. It can be deduced from our work that changes made in the ingredients of GF foodstuffs not only had the aim of improving the technological properties of pasta but also of adapting to the market demands and enhancing the final product by offering lower lipid content.

### 3.2. Nutritional Composition of GF Products vs. Homologous Foods with Gluten

The results of the present study comparing the nutritional profile differences between GF-rendered foodstuffs and their non-GFP counterparts in 2022 identified significant differences. The data currently obtained showed overall major changes in the following variables: a significantly lower protein content was observed in GFP compared to GCP (4.46 ± 3.01 against 8.51 ± 2.99; *p* ≤ 0.001) and a substantial increase in salt (1570 ± 6317 vs. 871 ± 993; *p* ≤ 0.16) and total carbohydrate content as well (64.6 ± 17.9 vs. 59.6 ± 14.8; *p* ≤ 0.001). These results were in line with a previous study by Babio et al. [22] since they also found that GFPs had a lower protein content compared to their gluten-containing counterparts. As expected, the lower protein content of GFPs is the outcome of the ingredients used in the formulation, such as corn starch and corn flour and rice flour, which naturally have a high-carbohydrate and low-protein profile [23]. Additionally, our results are in agreement with similar studies that attested that GFPs, compared to homologous GCPs, contain lower protein and higher carbohydrate and salt contents [4,23].

Mirroring the previous analysis of GFPs in two different periods, energy, nutrients, and fiber values for GFP and their non-GFP counterparts were represented in Table 2, organized by food groups. Significantly lower protein content was observed in GFPs in the categories of cookies (−71.5%) (*p* ≤ 0.001, r = −0.60), pastries (−63.1%) (*p* ≤ 0.001, r = −0.59), pasta (−64.5%) (*p* = 0.03, r = −0.64), doughs, puff pastry and pizzas (−268%) (*p* = 0.03, r = −0.64), and breads (−179%) (*p* ≤ 0.001, r = −0.62) compared to non-GF foodstuffs. These results are consistent with our previous study in 2013 [9] in which GF breads had almost a third less protein than their equivalent with gluten, and also with Jamieson et al. [24], who showed that their GFPs (bread, cookies, cereals, and pasta) obtained 36% less protein than their GCPs. Overall, this might reflect the impact of gluten proteins on the overall protein content in gluten-containing food.

Regarding total carbohydrate content, it was significantly higher across all the GFPs in the categories of cookies (+71.5%), pastries (+2.28), pasta (+7.95%), baby food (+17.7%), doughs/puff pastry/pizzas (+21.2%), and breads (+9.87%) compared to homologous GCPs. Similarly, several authors claimed a higher content of carbohydrates for GFP in their studies [25,26,27]. Furthermore, as explained by Lavriša et al. [28], GF ingredients used as gluten substitutes are usually carbohydrate-based ingredients, such as corn or potato starch and refined GF flours.

Over the last nine years, from the publication of our first study to the present, there have been variations in the nutritional profile of GFPs vs. GCPs. In fact, pasta and bread were the groups that have undergone the most relevant changes. Different research groups worldwide have undertaken studies on the nutritional profile of these foodstuffs available on the market based on their labels. Hence, it was interesting to make a temporary analysis of these reports from previous years. In our first research back in 2014 [9], with food data collected between 2012 and 2013 (Spain, *n* = 206 GFP and *n* = 289 GCP), the category of GF breads presented a lower content of protein and fiber and a higher content of saturated lipids and salt than their equivalents with gluten. Equally, the group of GF pasta had a nutrient profile similar to GF bread. Kulai and Rashid [25], with a database of Canadian foods from 2012 to 2013 (*n* = 168 GFP and *n* = 162 GCP), reported higher fat but lower protein in GF breads. The mean carbohydrate content of GF pasta was higher whereas protein, sugars, and fiber were lower. Later on, Wu et al. [29] showed in their Australian food data collected in 2013 (*n* = 173 GFP and *n* = 1006 GCP) that GFPs in the core food categories had similar nutritional profiles compared with non-GF products overall except for GF pasta and bread, whose average protein levels declined. The content of sugars and calories was also lower in the pasta group. Austrian food data from 2014 and 2015 by Missbach et al. [30] (*n* = 63 GFP and *n* = 126 GCP) reported that pasta and bread clusters exhibited significantly minor protein levels while carbohydrates, sugars, and fiber were higher in the GF pasta, and GF breads provided a lower salt content. In general, it can be concluded that GF pastas had a lower amount of protein, as demonstrated by Kulai et al. [25], Wu et al. [29], Missbach et al. [30], and Miranda et al. [9]. However, data for carbohydrate and fiber content were contradictory. The Kulai et al. [25] and Missbach et al. [30] studies showed an increase in the carbohydrate content of GF pasta. The Wu et al. [29] study, nonetheless, indicated no changes. Fiber levels were notably lower in the results of the Kulai et al. [25] and Miranda et al. [9] studies; nevertheless, Missbach [30] presented high values, and Wu did not describe changes in this aspect. In the bread category, most of the studies cited reported a notably lower protein content with the exception of Kulai et al. [25]. The rest of the nutrients, including lipids, salt, and carbohydrates, presented high variability. Likewise, there were important differences due to the different sample sizes, which in the case of GFPs, ranged from 63 to 203. The discrepancies might also be due to the fact that the studies were developed in different countries and continents, and as affirmed by Toledo et al. [31], food production and consumption need to continuously adapt to the changing demands of society, and biodiversity provides the basis to confront change.

Since time is a highly significant variable and GFPs can change through the years, it is interesting to search for the most recent studies regarding these products. When analyzing studies of the last four years, it is also possible to observe important variations in the nutritional profile of GFPs and their equivalents, particularly in the bread and pasta categories. Previously, Allen and Orfila [32], in their British food data collection from 2017 (*n* = 49 GFP and *n* = 61 GCP), reported that GF breads had significantly increased in fat and fiber content. However, carbohydrate and protein levels were lower both in GF white bread and GF pasta when compared to standard products. The fiber content of GF pasta was similarly lower as well. Subsequently, Calvo-Lerma et al. [23], with a database of Spanish foods from 2017 (*n* = 621 GFP and *n* = 600 GCP), pointed out that breads had a higher content of total and saturated fat, and GF pasta revealed reduced quantities of protein and sugar when compared with their homologous products. Later on, another Spanish study with data collected in 2018 (*n* = 358 GFP and *n* = 358 GCP) described significantly minor amounts of protein and sugars in GF pasta and bread [21]. Moreover, these two groups also presented punctual decreasing energy values while fiber increased in both. The salt content was major in pasta and inferior in bread. Regarding the pasta group, in our present study, we obtained enhanced energy and carbohydrate content. Conversely, protein levels were significantly lower. The group of breads also obtained markedly reduced protein but higher levels of carbohydrates and sugars.

In summary, regarding the GF pasta category, it can be concluded that protein levels were diminished as reported by others [21,23,32] and our present results. Likewise, energy and sugars were also lower [21,23] as in the present research. Given the fact that most of the research groups are Spanish, it is striking that lower levels of sugars and energy tend to be used in our country while in the United Kingdom, they did not share the same practice. Considering the above results, it is possible to deduce that the country where the food is produced was a variable to take into account for the evaluation of GF pasta. Regarding fiber content in the pasta group, there were inconsistencies since Allen and Orfila [32] and our present study reported low levels, De Las Heras-Delgado et al. [21] reported higher rates, and Calvo-Lerma et al. [23] did not observe changes. It should be noted that, similarly, the present research found that GF pasta had a higher salt content than GC pasta, something that was also reported in another study of Spanish origin on pasta [21]. The GF bread category showed reduced protein levels in the present study. This is in line with other authors [21,32] despite the fact that Calvo-Lerma et al. [23] found no changes when comparing GF vs. GC breads. In addition, the lipid content was higher according to our studies and others [23,32] but not to De las Heras-Delgado et al. [21]. We described an increase in total and simple carbohydrates in GF products compared to GC food while Allen and Orfila [32] reported decreased values of total carbohydrates, and De Las Heras–Delgado et al. [21] showed a significant decline in the content of sugars. In addition, De Las Heras-Delgado et al. [21] were the only researchers who detected varying levels of salt in the category of GF breads.

GF breads thereby seem to share the characteristics of less protein and more fat compared to GC breads. For the rest of the parameters, the heterogeneity of the results did not allow a general statement to be framed. Perhaps this may be because the group of breads can be organized into numerous subgroups due to its heterogeneity and according to different criteria varying according to the researcher’s viewpoint or the study design (for example, bread toast, bread rolls, baguettes, bread buns, etc.).

### 3.3. Comparison between a Diet with GF Foods and a Diet with Equivalent Products with Gluten

Nowadays, the only therapy available that has been accepted for CD is adherence to a GF diet. It is well known that commercial GFPs often have compromised nutritional quality compared to their gluten-containing equivalents [33]. The use of gluten-free products and diets by people not diagnosed with celiac disease has become widespread. As the diet has gained popularity from media coverage and celebrity promotion, many people have adopted the diet despite not having any gluten-related disorder. For these individuals, gluten avoidance may cause nutritional deficiencies which could otherwise be prevented [34]. Taking this into account and in order to emphasize the shortfalls or excesses that could be caused by following a GF diet, a comparison between a GF diet and the same diet with gluten foodstuffs (a simulated diet) was carried out.

In the present study, following a GF diet, the results concerning energy distribution reported no significant differences between the gender of the participants (data not shown). However, there were variations in favor of the percentage of carbohydrates in total energy intake, which was boosted by following a GF diet (Table 3). This is in accordance with Lavriša et al. [28], who detected that GF ingredients which are used as gluten substitutes are usually carbohydrate-based ingredients, such as corn or potato starch and refined GF flours, and this is consistent with the fact that GFPs tended to have a higher glycemic index than their gluten-containing counterparts [1,35]. It was demonstrated that the risk of obesity is increased in CD people on a GF diet because of the high glycemic index of the GF diet [36]. Furthermore, information obtained in the present study also suggested that a GF diet was related to a lower energy intake from proteins. Taking into account that gluten is the main protein in various foods from the group of cereals, such as wheat, barley, and rye, their entire elimination in the manufacturing of products leads to a reduction in the overall protein intake [21]. As expected, the lower protein content of GFPs is the result of the ingredients used in the formulation, such as corn starch and corn flour and rice flour, which naturally have a high-carbohydrate and low-protein content [23]. Nevertheless, it is important to bear in mind that the dietary habits of the celiac population compared to the general one showed a lower consumption of cereal-containing foods and, as a result, lower carbohydrate intake [37]. The higher content of this macronutrient in GFPs seems to ameliorate that imbalance.

Several studies have evaluated the dietary adequacy and nutritional status of patients with celiac disease. Currently, there is controversy over the nutritional balance between a GF diet and a regular gluten-containing diet [22]. As a matter of fact, our research group detected nutritional deficiencies in GF diets. A challenge for food manufacturers could be considering fortifying GF foods to achieve an improved nutritional quality of processed GF foods. We highlight that in the era of functional foods with a constantly increasing demand, GFPs are a target for innovation. More attention should be paid to improving GFP composition since an extensive number of studies have been performed focusing on alternative, healthy, and nutritionally rich ingredients for GFP formulation [23]. Fortification of GF foods could also compensate for possible micronutrient deficiency in CD patients [28] and macronutrient insufficiency as well as compensate nonceliacs who consume GF-rendered foods.

### 3.4. Strengths and Limitations

As this research is a continuation of a previous investigation, the strengths of this study were that we could observe the nutritional changes that have occurred in the composition of GFPs in comparison with their homologous products over a period of time. In our past study [9], there were significant changes in the total energy intake, and percentages of protein, carbohydrates, and fiber decreased in those patients following a GF-processed food diet. In the current investigation, we did not obtain significant variations in the percentage of fiber or fat. Perhaps Spanish companies are making an effort to improve the nutritional profile of their GFPs [22]. Some limitations of this study need to be recognized. Nutritional information was obtained through food labeling instead of chemical analysis, which is a specific additional procedure; therefore, micronutrient information was not studied in this research. A second limitation is the low number of GF brands used for nutritional comparison against the conventional gluten brands. Due to the careful exclusion steps applied in this research, 104 of the originally identified 206 GF products were analyzed, and only packaged food products were sampled. Consequently, findings cannot be extended to the overall nutritional quality of an everyday GF diet that may include home-cooked meals with fresh, natural GF ingredients as stated by Kulai et al. [25]. In addition, our data is unable to generalize about other countries, as this research has only considered Spanish products.

## 4. Conclusions

It can be concluded that reviewing the nutritional composition of GF foods from time to time is highly relevant since these products, which are in great demand, undergo constant changes in their composition with the aim of improving their nutritional quality. Despite improvements in the formulation of GFPs in recent years, their macronutrient profile suggested they contained marked differences and cannot be considered nutritionally equivalent when compared with their gluten-containing counterparts. Therefore, it is strongly recommended that food companies continue with the reformulation of these products in order to increase their nutritional quality, adapt to market demands, and accordingly provide balanced nutrition to those patients with CD.

## Figures and Tables

**Table 1:** Energy, protein, total carbohydrates, simple carbohydrates, total lipids, and saturated lipids content per 100 g of products for gluten-free rendered foodstuffs classified by food groups in 2013 and 2022.

Content/Foodstuff		Cookies				Pasta				Breakfast Cereals				Dough, Puff Pastry, Pizza				Breads			
	Year	M	SD	*p*	r	M	SD	*p*	r	M	SD	*p*	r	M	SD	*p*	r	M	SD	*p*	r
Energy (kcal)	2013	481	68.8	0.38	−0.16	365	12.5	0.02	−0.65	381	22.5	0.58	−0.14	275	62.5	0.35	−0.27	324	75.3	0.83	−0.03
	2022	488	35.8			361	5.81			386	24.5			350	89.2			323	74.1		
Protein (g)	2013	5.02	1.73	0.02	−0.43	8.38	0.98	0.03	−0.61	8.65	2.09	0.35	−0.23	1.22	1.02	0.27	−0.32	3.81	2.44	0.67	−0.05
	2022	4.18	1.14			7.78	0.73			8.26	2.20			1.85	1.46			3.66	2.07		
Total carbohydrates (g)	2013	65.8	9.06	0.96	−0.01	75.6	2.95	0.91	−0.03	76.7	10.1	0.87	−0.04	57.4	24.8	0.23	−0.35	60.9	15.3	0.25	−0.14
	2022	67.9	5.55			75.5	0.84			76.6	8.85			67.0	16.5			62.8	16.2		
Simple carbohydrates (g)	2013	26.2	6.76	0.87	−0.03	0.40	0.00	0.03	−0.65	15.2	10.4	0.50	−0.17	12.6	15.3	0.50	−0.19	4.48	2.62	0.61	−0.06
	2022	26.6	8.10			0.37	0.16			16.0	8.12			10.5	15.9			5.02	4.23		
Total lipids (g)	2013	21.6	6.15	0.44	−0.14	4.53	4.20	0.34	−0.28	3.78	3.37	0.87	−0.04	3.32	3.54	0.69	−0.12	6.50	5.34	0.11	−0.19
	2022	21.5	6.37			2.67	0.89			5.22	5.03			7.71	13.8			5.21	4.52		
Saturated lipids (g)	2013	10.3	5.61	0.47	−0.13	1.02	0.27	0.05	−0.58	1.46	1.28	0.08	−0.44	1.14	1.62	0.69	−0.12	2.58	2.55	0.02	−0.29
	2022	10.7	7.39			0.57	0.46			1.25	1.29			3.47	7.42			1.80	2.44		
Dietary Fiber (g)	2013	2.65	1.12	0.27	−0.20	2.72	1.16	0.03	−0.61	4.39	1.54	0.69	−0.10	1.99	1.26	0.04	−0.59	3.97	2.43	0.17	−0.17
	2022	3.65	1.95			2.08	0.18			4.86	1.97			2.78	1.29			4.58	3.04		
Salt (mg)	2013	452	468	0.12	−0.28	208	102	0.04	−0.59	886	885	0.61	−0.13	917	904	0.46	−0.21	1502	686	0.29	−0.13
	2022	493	288			38.3	72.2			829	827			1090	637			1407	537		

Values are expressed as means (M) and standard deviations (SD).

**Table 2:** Energy, protein, total carbohydrates, simple carbohydrates, total lipids, and saturated lipids content per 100 g of products in gluten-containing and gluten-free rendered foodstuffs classified by food groups.

Content/Foodstuff		Cookies				Pastries				Pasta				Baby Food				Dough, Puff Pastry, Pizza				Breads			
		M	SD	*p*	r	M	SD	*p*	r	M	SD	*p*	r	M	SD	*p*	r	M	SD	*p*	r	M	SD	*p*	r
Energy (kcal)	GF	488	35.8	0.92	−0.02	400	73.8	0.92	−0.02	361	5.81	0.04	−0.59	451	159	0.27	−0.29	350	89.2	0.89	−0.04	323	74.1	0.78	−0.03
	GC	486	35.6			448	49.6			353	8.65			386	17.2			388	128			319	68.1		
Protein (g)	GF	4.18	1.14	<0.001	−0.60	3.58	2.52	0.00	−0.59	7.78	0.73	0.03	−0.64	8.56	5.47	0.13	−0.41	1.85	1.46	0.03	−0.64	3.66	2.07	0.00	−0.62
	GC	7.17	1.62			5.84	1.62			12.8	0.99			11.2	3.23			6.80	1.67			10.2	2.49		
Total carbohydrates (g)	GF	67.9	5.55	0.01	−0.49	48.3	5.28	0.01	−0.47	75.5	0.84	0.03	−0.64	90.3	20.8	0.04	−0.54	67.0	16.5	0.03	−0.64	62.8	16.2	0.01	−0.33
	GC	63.2	8.08			49.4	4.88			69.5	2.29			74.3	5.67			52.8	17.9			56.6	14.9		
Simple carbohydrates (g)	GF	26.6	8.10	1.00	0.00	19.1	8.18	1.00	0.00	0.37	0.16	0.03	−0.64	24.7	21.4	0.31	−0.27	10.5	15.9	0.35	−0.27	5.02	4.23	0.02	−0.29
	GC	23.9	11.7			20.8	9.86			2.83	0.84			11.8	12.6			2.58	1.38			3.60	2.17		
Total lipids (g)	GF	21.5	6.37	0.43	−0.14	22.8	6.45	0.43	−0.14	2.67	0.89	0.03	−0.64	5.70	8.22	0.50	−0.18	7.71	13.84	0.92	−0.03	5.21	4.52	0.32	−0.12
	GC	22.5	7.03			24.8	6.41			2.00	0.84			3.60	2.63			6.67	8.34			4.28	3.65		
Saturated lipids (g)	GF	10.7	7.39	0.76	−0.06	7.89	5.17	0.76	−0.05	0.57	0.46	0.52	−0.19	1.98	3.58	0.69	−0.11	3.47	7.42	0.75	−0.09	1.80	2.44	0.04	−0.25
	GC	9.90	6.25			9.26	7.39			0.48	0.30			0.64	0.66			2.62	4.22			0.74	0.56		
Dietary Fiber (g)	GF	3.65	1.95	0.33	−0.17	2.14	1.34	0.33	−0.17	2.08	0.18	0.04	−0.59	2.32	1.89	0.03	−0.59	2.78	1.29	0.03	−0.64	4.58	3.04	0.09	−0.21
	GC	2.88	2.79			1.31	1.43			3.46	0.74			5.87	2.72			0.37	0.90			3.85	3.48		
Salt (mg)	GF	493	288	0.74	−0.06	1136.5	477	0.74	−0.06	38.3	72.2	0.33	−0.28	597	726	0.14	−0.39	1090	637	0.46	−0.21	1407	537	0.09	−0.20
	GC	494	329			693	316			50.5	40.9			194	401			1535	622			1221	800		

Values are expressed as means and standard deviations (SD). Gluten-free (GF); Gluten-containing (GC).

**Table 3:** Macronutrient (carbohydrates, proteins, and lipids) percentage from the energy intake and fiber intake (g/d) for males and females following a diet with gluten-free rendered foods and a diet with equivalent products with gluten.

Content		Mean	SD	*p*	r
Carbohydrates % from the total energy intake	GF	40.8	7.29	0.001	−0.460
	GC	40.0	6.88		
Lipids % from the total energy intake	GF	42.1	7.54	0.904	−0.017
	GC	42.2	7.53		
Proteins % from the total energy intake	GF	17.0	3.49	<0.001	−0.592
	GC	17.9	3.57		

Values are expressed as means and standard deviations (SD). Gluten-free (GF); Gluten-containing (GC).

## Data Availability

The data described in this manuscript will be made available upon request pending application and approval by the corresponding author.
